# The impact of Yoga on patients with knee osteoarthritis: A systematic review and meta-analysis of randomized controlled trials

**DOI:** 10.1371/journal.pone.0303641

**Published:** 2024-05-16

**Authors:** Junyue Lu, Jiliang Kang, Haoyuan Huang, Chen Xie, Jiaxuan Hu, Yan Yu, Yu Jin, Youliang Wen

**Affiliations:** 1 School of Rehabilitation Medicine, Gannan Medical University, Ganzhou, Jiangxi, China; 2 The Third Affiliated Hospital of Gannan Medical University, Ganzhou, Jiangxi, China; Sheikh Hasina National Institute of Burn & Plastic Surgery, BANGLADESH

## Abstract

**Objective:**

The objective of this review is to conduct a comprehensive and systematic assessment of the efficacy of Yoga as an intervention for knee osteoarthritis (KOA).

**Methods:**

We searched PubMed, Cochrane Library, Embase, Web of Science, and PEDro as of January 3, 2024. Retrieved a total of 200 articles. Standardised mean differences (SMDs) and 95% confidence intervals (CI) were calculated.

**Results:**

The study included a total of 8 trials and involved 756 KOA patients. The results indicated that compared to the control group, Yoga exercise showed significant improvements in alleviating pain (SMD = -0.92; 95% CI = -1.64 ~ - 0.20; *P* = 0.01, *I*^2^ = 94%), stiffness (SMD = -0.51; 95% CI = -0.91 ~ -0.12; *P* = 0.01; *I*^2^ = 66%) and physical function (SMD = -0.53; 95% CI = -0.89 ~ -0.17; *P* = 0.004; *I*^2^ = 59%) among KOA patients. However, there was no significant improvement observed in terms of activities of activity of daily living (ADL) (SMD = 1.03; 95% CI = -0.01 ~ 2.07; *P* = 0.05; *I*^2^ = 84%), and quality of life (QOL) (SMD = 0.21; 95% CI = -0.33 ~ 0.74; *P* = 0.44; *I*^2^ = 83%) with the practice of Yoga.

**Conclusions:**

In general, Yoga has been found to be effective in reducing pain and stiffness in KOA patients, it can also improve the physical function of patients. However, there is limited evidence to suggest significant improvements in terms of ADL and QOL.

## 1 Introduction

Osteoarthritis (OA) is a common joint disease characterized by the degeneration and damage of joint cartilage, often accompanied by joint pain and stiffness. Knee osteoarthritis (KOA), which is the most common type of OA, has a higher incidence in females than males [1, 2]. It is more prevalent among individuals with unhealthy lifestyles and obesity. Statistics show that nearly one-third of middle-aged and elderly individuals over 50 years old suffer from KOA, which can potentially progress and lead to lifelong disability [3, 4]. The course of KOA is chronic and slow, primarily characterized by pain, stiffness, and impaired physical function [5]. It significantly affects daily activities such as climbing stairs and squatting. Severe cases may experience intense pain even during walking, further impacting the physical and mental health as well as the quality of life (QOL) of patients [6, 7].Currently, there are three main categories of standardized treatment methods for KOA: pharmacological treatment, nonpharmacological treatment, and surgical treatment [8].

Pharmacological treatments for KOA include nonsteroidal anti-inflammatory drugs, such as and acetaminophen, which have varying efficacy and can only provide short-term relief of inflammation and pain [9, 10]. There are also long-term medications like tricyclic antidepressants such as amitriptyline and opioids. These medications may exert certain pressures on organs such as the liver, kidneys, and gastrointestinal tract. Surgical treatment is generally considered as a last resort for patients and carries certain risks, especially for middle-aged and elderly individuals [11, 12]. Older adults often have multiple underlying conditions such as hypertension, diabetes, and heart disease, which may increase the surgical risks [13]. Additionally, the physical function of older individuals tends to decline, leading to slower postoperative recovery that requires longer periods of rehabilitation and care [14]. Therefore, non-pharmacological therapies such as exercise, physical therapy, and massage are more readily accepted by patients in clinical practice. Research has shown that nearly 80% of OA patients lack physical activity, which is a major contributing factor to poor prognosis and accelerated progression of the disease [15, 16]. Exercise serves as a core nonpharmacological treatment for KOA and offers advantages such as safety, environmental friendliness, effectiveness, and low cost. Aerobic exercises like walking, cycling, swimming, Yoga, tai chi, and Ba Duan Jin, as well as strength training and balance training, are commonly used to treat KOA [17–19].

Yoga originated in ancient India and has become a popular form of exercise in recent years [20]. Approximately one-fifth of American adults use Yoga to maintain their health [21]. Unlike jogging or walking, Yoga movements are slow, gentle, and sustained, making it suitable for middle-aged and elderly people, including those with KOA [22]. Yoga encompasses various styles, such as Iyengar Yoga and Hatha Yoga, combining different postures, breathing exercises, and meditation elements [23]. Hatha Yoga emphasizes the control of breathing and posture, achieving physical balance and coordination through practice. Iyengar Yoga places more emphasis on balancing and coordinating the body, mind, and spirit [24, 25]. Practicing meditation in Yoga can help relax the body and mind, reduce psychological stress, and improve sleep quality and emotional state. In addition, studies have shown that meditation can reduce pain sensitivity and induce analgesic effects. Therefore, Yoga provides a comprehensive approach to managing KOA, providing physical benefits through stretching and strengthening, as well as psychological benefits through relaxation and stress reduction [26, 27].

However, current clinical research on Yoga for KOA has utilized different outcome measurement methods and varied sample sizes in the trials. There has been a persistent lack of systematic reviews and meta-analyses specifically examining randomized controlled trials (RCTs) on the use of yoga for treating patients with KOA. Therefore, our systematic review and meta-analysis rigorously included studies on KOA patients and employed RCT designs to investigate the therapeutic effects of Yoga in this population.

## 2 Methods

### 2.1 Protocol and registration

This meta-analysis was planned and conducted according to the PRISMA guidelines and the Cochrane Handbook for Systematic Reviews of Interventions [28, 29]. The complete protocol of this meta-analysis was uploaded and registered on the PROSPERO platform with the registration number: CRD42024500240. As all the data in this study were obtained from experimental articles and no direct recruitment or collection of patient information was involved, ethical approval or consent statement was not required.

### 2.2 Literature search

A total of 5 databases (PubMed, EMbase, PEDro, Web of Science, and Cochrane Library) were searched for trials related to the use of Yoga for the treatment of KOA from the inception of the databases until January 1, 2024 (The detailed PubMed retrieval process is shown in S2 File). There were no language restrictions imposed during the search. The search terms used were "Yoga" and "knee osteoarthritis." Two researchers (Lu and Kang) conducted the search independently, and in case of any discrepancies, a third researcher (Wen) reviewed the divergent results to make the final decision. Please refer to S1 File for the search formula and search history.

### 2.3 Study selection

All the retrieved records were screened by researchers (Lu and Kang) simultaneously during the same time period. They documented all the included and excluded records along with the reasons for exclusion [30]. Any issues that arose during the screening process were discussed separately with researcher (Wen). In case of any discrepancies in the final results, researcher (Wen) conducted a review and a meeting was held with all three researchers to reach a consensus before finalizing the decisions.

The inclusion criteria were as follows: (1) Only patients with KOA were included, diagnosed based on the clinical and radiological criteria defined by the American College of Rheumatology (ACR) and the Kellgren-Lawrence grading of osteoarthritis severity. (2) The intervention group could receive adjunctive therapies, but these therapies should not include various forms of exercise therapy. The control group must also receive these adjunctive therapies, or alternatively, Yoga must be the sole variable. There were no restrictions on the type of Yoga. (3) The study design had to be a randomized controlled trial (RCT). (4) At least one of the following outcome measures was required: pain, stiffness, physical function, activity of daily living (ADL) or QOL.

Exclusion criteria: (1) Studies with incomplete data. (2) The data is presented in the form of a graph. (3) Incomplete description of the trial protocol. (4) Randomized crossover study design. (5) Research published in non-English language.

### 2.4 Data extraction and management

The data extracted from the included literature for analysis include: author and publication year, country of the trial, patient age, intervention methods for the intervention and control groups, type of Yoga in the intervention group, intervention period and frequency, duration of each treatment session and outcome measures. This process was independently conducted by researchers (Lu and Kang), who created a table for data extraction. Once completed, the extracted results were cross-checked, and any issues were reviewed and corrected by researcher (Huang). The final version of the table and data were determined after discussion and consensus among all three researchers.

### 2.5 Quality assessment

Two researchers (Lu and Kang) independently assessed the risk of bias using Review Manager 5.4, and objectively evaluated the risk of bias in the included literature by reading the full text. The assessment included the following domains: selection bias (random sequence generation, allocation concealment), performance bias (blinding of participants and personnel), detection bias (blinding of outcome assessment), attrition bias (incomplete outcome data), and reporting bias (selective reporting). Each domain was evaluated and categorized into three levels: (1) low risk of bias, (2) high risk of bias, and (3) unclear. After completing their assessments, the two authors found only one discrepancy. After discussing with author (Hang), a consensus was reached.

### 2.6 Data analysis

All the data used in this study were analyzed and processed using the meta-analysis software Review Manager 5.4 from the Cochrane Collaboration. Among the included studies, there were multiple evaluations of outcomes at different stages of the trials. We selected the results that were closest to 8 weeks for our analysis. All the measured values were continuous variables, and therefore, the standardized mean difference (SMD) was used to calculate the differences. The z test was used to evaluate the 95% confidence interval (CI). Heterogeneity between groups was tested by the Cochran’s Q statistics and I^2^ test. If there was no heterogeneity between the groups (Q test shows *P*>0.05 or *I*^2^<50%), a fixed-effect model would be applied. Otherwise, if the Q test results were significant (*P*<0.05 or *I*^2^>50%), a random-effect model would be used in the meta-analysis [31]. In cases where standard deviations were not reported, they were calculated based on standard errors, CI, or t-values. Statistical heterogeneity between each citation was determined and quantified by the *I*^2^ parameter. An *I*^2^ value of 50% or higher was considered an indicator of substantial heterogeneity. When *I*^2^ >50%, perform subgroup analysis and sensitivity analysis to identify the source of heterogeneity and provide explanations. If there is insufficient sample size for subgroup analysis, proceed with sensitivity analysis only. *P* < 0.05 were considered to be statistically significant.

## 3. Results

### 3.1 Search results

**Fig 1:**
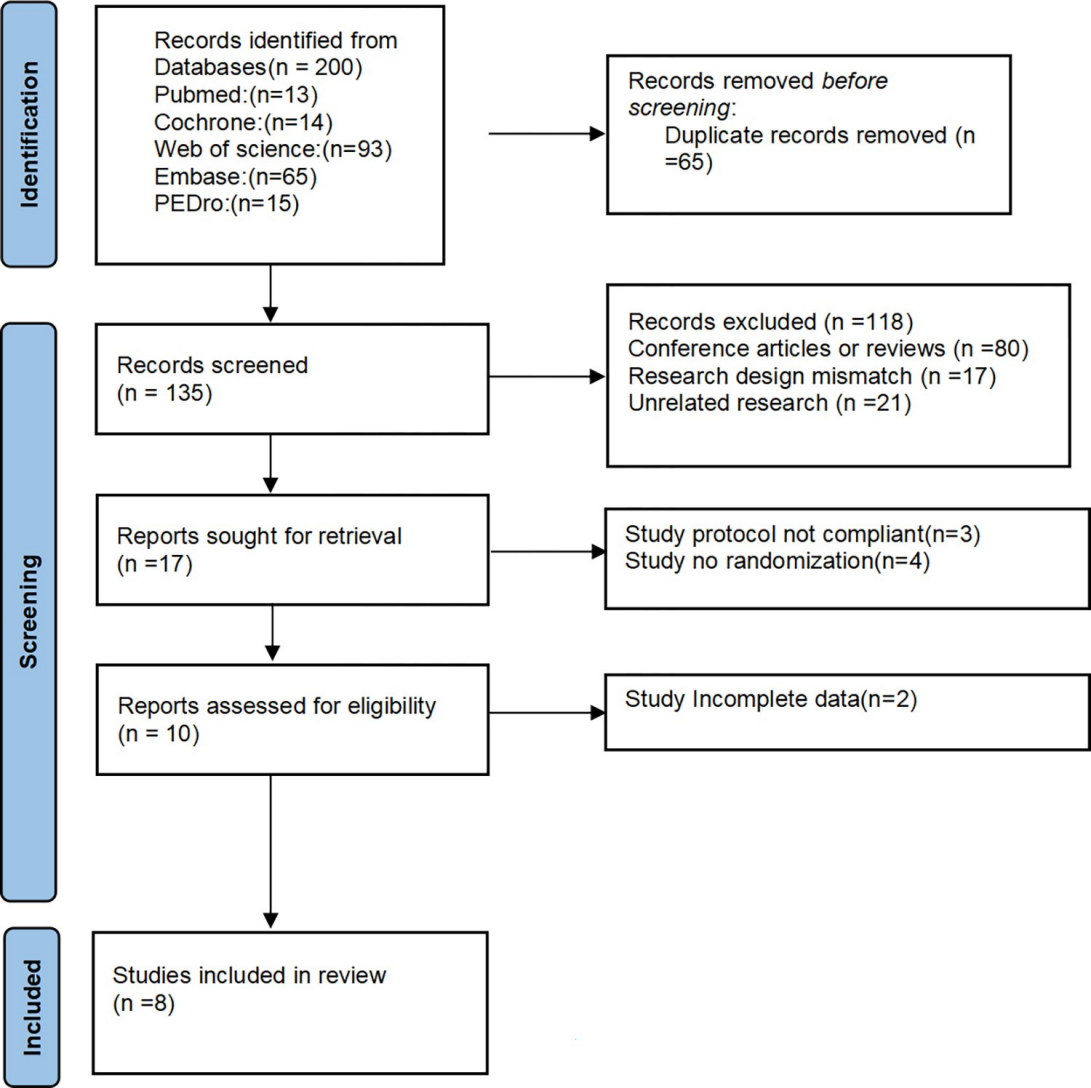
Flow diagram of included studies.

The entire process of literature search and screening is illustrated in Fig 1. A total of 200 relevant records were retrieved from five commonly used medical databases (PubMed = 13, Cochrane = 14, Web of Science = 93, Embase = 65, PEDro = 15). All records were imported into EndNote for management. Initially, 65 duplicate articles were excluded, resulting in 135 remaining articles. Following the PICO format, the titles and abstracts were reviewed, leading to the exclusion of 80 review articles or conference abstracts, 17 articles with study designs incompatible with controlled groups or pre-post comparisons, and 21 articles unrelated to rheumatoid arthritis or arthritis in other joints. After excluding the aforementioned 118 articles based on their titles and abstracts, the full text of 17 articles was reviewed. Three articles were excluded as their study protocols did not meet the criteria, and an additional four articles were excluded due to lack of randomization. During the data extraction phase, two studies were excluded due to incomplete data. A total of 8 studies were included in this article (The literature excluded after reading the full text is detailed in S3 File).

### 3.2 Study characteristics

Table 1 shows the baseline characteristics of patients included in the 8 randomized controlled trials. These studies were published between 2012 and 2023 and included a total of 756 KOA patients. All 8 studies were published in English, with 2 conducted in India [32, 33], 2 in the United States [34, 35], and 1 each in China [36], Canada [37], Iran [38], and Australia [39]. In 2 studies [32, 33], the intervention group received Yoga in combination with other treatments. Among the 5 studies that specified the type of Yoga used, Hatha Yoga was employed in 4 studies [34, 35, 38, 39], Chair Yoga in 1 study [36], and the remaining 3 studies did not explicitly mention the type of Yoga or utilized a combination of different types. Six studies assessed pain as an outcome measure [32–35, 38, 39], while four studies assessed physical function [33–35, 39]. Additionally, four studies evaluated knee joint stiffness [33–35, 39], three studies evaluated activities of ADL [36–38], and six studies assessed QOL [33–35, 37–39]. Although different assessment scales were used across these studies, the data related to the same outcome measures were analyzed together.

**Table 1:** Basic characteristics of included citations.

Author, year	Country	E/C (N)	Age(year) (M±SD)	Experimental group	Control group	Yoga type	Time/times/weeks of yoga	Outcome
Yao,2023	China	43/42	E:76.37±6.08 C:78.66±6.18	Yoga	Community activities	Chair yoga	110min/2/w/10w	ADL
Kuntz,2018	Canada	10/10	E:65.5±5.6 C:63.7±8.9	Yoga	Sessions	-	60min/3/w/12w	KOOS
Ghasemi,2012	Iran	15/15	E:51±8.9 C:53.11±10.9	Yoga	Daily activities	Hatha yoga	60min/3/w/8w	VAS,ADL,QOL
Ebnezar,2012	India	118/117	E:59.56±8.18 C:59.42±10.66	Yoga+Conventional physiotherapy	Non-yogic exercises+Conventional physiotherap	-	40min7/w/12w	VAS, WOMAC
Cheung,2014	USA	18/18	E:71.9±3.93 C:71.9±4.44	Yoga	Wait-list	Hatha yoga	60min/1/w/8w	WOMAC,SF-12
Cheung,2016	USA	32/23	E:68.9±7.7 C:71.8±8.0	Yoga	Education	Hatha yoga	45min/4/w/8w	WOMAC,VAS,SF-12,
Bennell,2022	Australian	105/107	E:62.8±8.2 C:61.8±7.2	Yoga	Education	Hatha yoga	30min/3/w/12w	WOMAC,QOL
Vaghela,2020	India	43/40	E:56.58±10.12 C:54.27±8.44	Yoga+Conventional physiotherapy	Conventional physiotherapy	-	40min/3/w/4w	WOMAC,SF-36

E = Experimental group; C = Control group; ADL = Activity of daily living; KOOS = Knee Osteoarthritis Outcome Score; VAS = visual analog scale; QOL = Quality of Life; WOMAC = Western Ontario and McMaster Universities Osteoarthritis Index; SF-12 = Health

related Short Form 12; SF-36 = Health related Short Form 36.

### 3.3 Quality assessment

**Fig 2:**
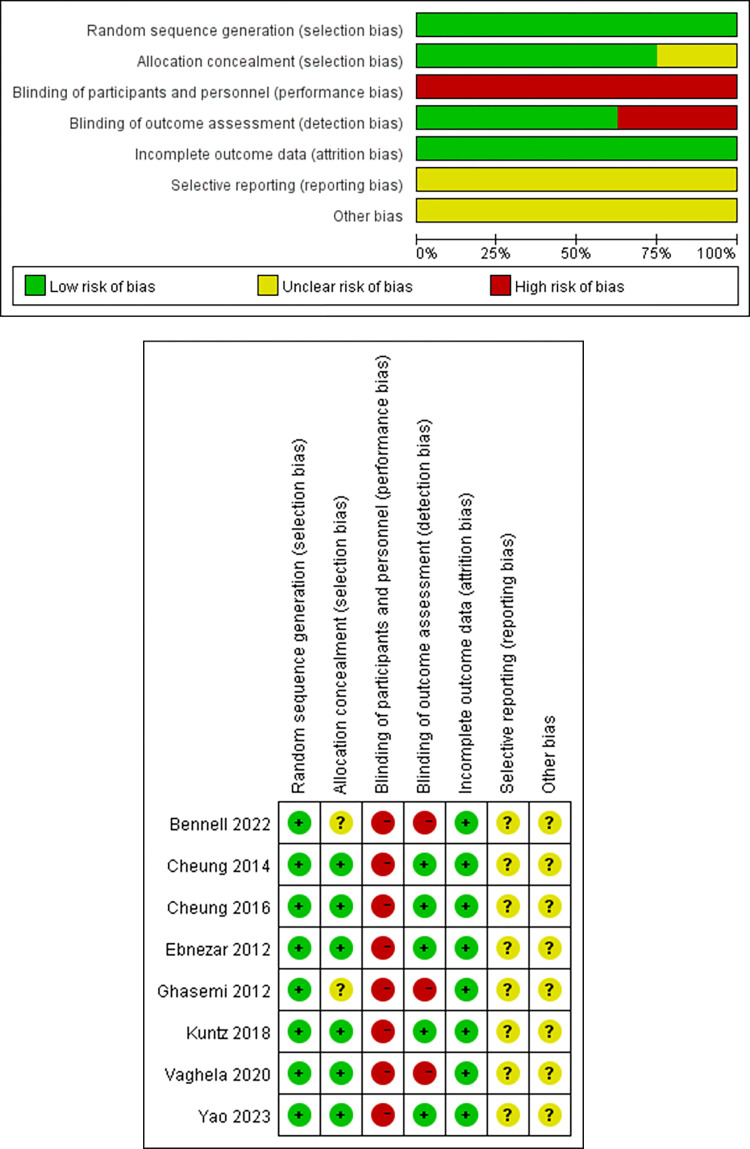
A. Risk of bias graph. B. Risk of bias summary.

Risk of bias assessments were conducted for all 8 included studies, as shown in Fig 2. All the included studies explicitly described their methods of randomization, which was one of our inclusion criteria (100%). Six studies implemented allocation concealment (75%) [32–37], while the remaining 2 studies did not provide information regarding allocation concealment in their publications [38, 39]. As the intervention involved Yoga treatment in the intervention group, blinding of the participants was not feasible (0%). However, 5 studies employed blinding for outcome assessors and data analysts (62.5%) [32, 34–37]. Incomplete outcome data (attrition bias) is all low-risk (100%). A cross all trials, the risk of selection bias was deemed unclear (100%). Due to the insufficient number of articles, we did not conduct a statistical analysis of publication bias.

#### 3.3.1 Yoga on pain

**Fig 3:**
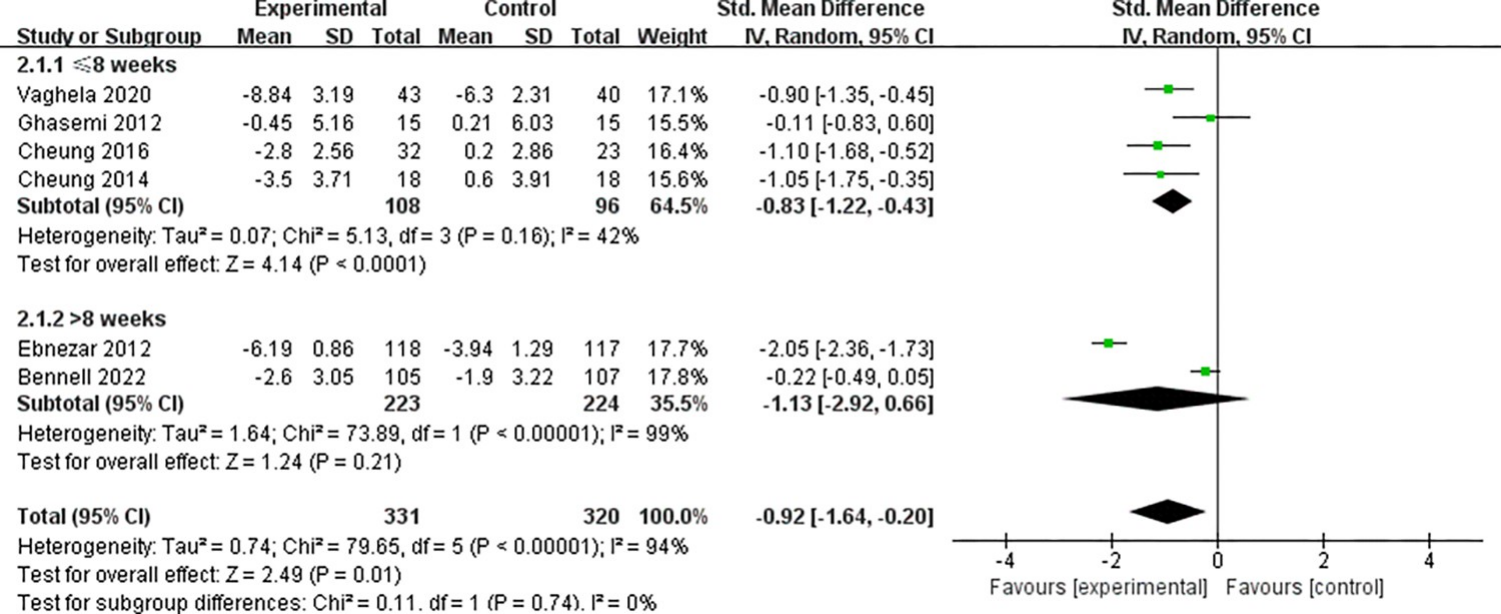
Yoga on pain.

A total of six studies [32–35, 38, 39] reported pain-related outcomes, involving 651 participants, as shown in Fig 3. Four studies [33–35, 39] used WOMAC (Western Ontario and McMaster Universities Osteoarthritis Index) to assess patient pain, while the other two [32, 38] used VAS (visual analog scale) for evaluation. The analysis results indicate that Yoga exercise can effectively alleviate pain in patients with KOA compared to the control group (SMD = -0.92;95% CI = -1.64 ~ - 0.20; *P* = 0.01, *I*^2^ = 94%).

To explore the sources of heterogeneity, we conducted a subgroup analysis based on the duration of pain intervention. This analysis categorized the studies into two subgroups: interventions with a duration of ≤8 weeks [33–35, 38] and interventions with a duration of >8 weeks [32, 39]. The subgroup analysis showed that in the four studies with a yoga intervention duration of ≤8 weeks, yoga significantly reduced pain in patients with KOA compared to the control group, with low heterogeneity (SMD = -0.83; 95% CI = -1.22 to -0.43; *P* < 0.0001; *I*^2^ = 42%). However, in the two studies with a yoga intervention duration of >8 weeks, there was no significant analgesic effect of yoga on KOA pain compared to the control group, and the heterogeneity was high (SMD = -1.13; 95% CI = -2.92 to 0.66; *P* = 0.21; *I*^2^ = 99%).

Furthermore, the subgroup analysis indicated that there was no significant difference in the analgesic effect of yoga between different intervention durations (≤8 weeks or >8 weeks) for KOA (*P* = 0.74). It is noteworthy that the overall analysis results of this study indicate that yoga is beneficial for pain relief in KOA compared to the control group. However, there is high heterogeneity (SMD = -0.92; 95% CI = -1.64 to -0.20; *P* = 0.01, *I*^2^ = 94%). This high heterogeneity may be attributed to the inclusion of the two studies with an intervention duration of >8 weeks [32, 39].

Due to high heterogeneity, we conducted sensitivity analysis on a case-by-case basis, as shown in Fig A in S4 File.

#### 3.3.2 Yoga on stiffness

**Fig 4:**
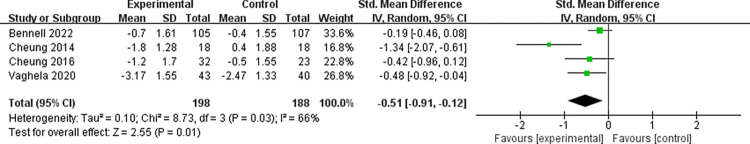
Yoga on stiffness.

Four studies [33–35, 39] evaluated the intergroup changes in knee joint stiffness in 386 patients, as shown in Fig 4. The results showed that compared to the control group, Yoga effectively reduced the severity of knee joint stiffness in patients with KOA (SMD = -0.51; 95% CI = -0.91 ~ -0.12; *P* = 0.01; *I*^2^ = 66%). After excluding the study [34] with the lowest weekly treatment frequency, we found that yoga still alleviated stiffness in patients with KOA, although heterogeneity decreased (SMD = -0.29; 95% CI = -0.50 ~ -0.08; *P* = 0.007; *I*^2^ = 0%), as shown in Fig B in S4 File.

#### 3.3.3 Yoga on physical function

**Fig 5:**
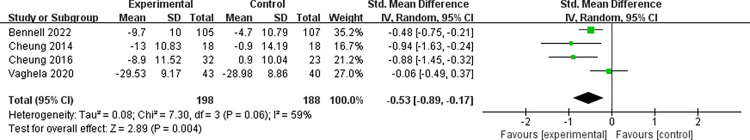
Yoga on physical function.

Four studies [33–35, 39] reported changes in physical function after Yoga treatment in 386 patients with KOA, as shown in Fig 5. Compared to the control group, yoga effectively improves the physical function of patients with KOA (SMD = -0.53; 95% CI = -0.89 ~ -0.17; *P* = 0.004; *I*^2^ = 59%). We found that one study [33] used a different type of yoga for training compared to the other three studies. After excluding this study, the results remained unchanged, but the heterogeneity significantly decreased (SMD = -0.65; 95% CI = -0.95 ~ -0.35; *P* < 0.0001; *I*^2^ = 24%), as shown in Fig C in S4 File.

#### 3.3.4 Yoga on ADL outcomes

**Fig 6:**
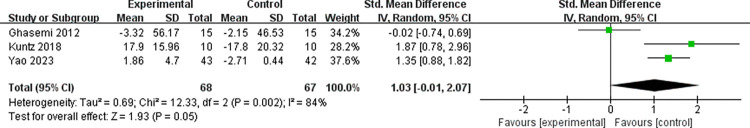
Yoga on ADL outcomes.

Data from 135 patients in three studies [36–38] were included in the analysis of ADL evaluation, as shown in Fig 6. The results showed that compared with the control group, the Yoga group had no significant improvement in daily activity (SMD = 1.03; 95% CI = -0.01 ~ 2.07; *P* = 0.05; *I*^2^ = 84%). After excluding a study with patients exhibiting milder symptoms [38], The results showed that Yoga had a significant improvement on the ADL in patients with KOA (SMD = 1.43; 95% CI = 1.00 ~ 1.87; *P* < 0.00001; *I*^2^ = 0%), as shown in Fig D in S4 File.

#### 3.3.5 Yoga on QOL outcomes

**Fig 7:**
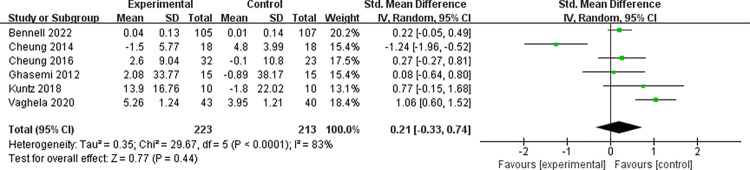
Yoga on QOL outcomes.

Six studies [33–35, 37–39] evaluated the QOL in 436 patients, as shown in Fig 7. The analysis results showed that there was no statistically significant difference in the change in QOL between the Yoga treatment group and the control group (SMD = 0.21; 95% CI = -0.33 ~ 0.74; *P* = 0.44; *I*^2^ = 83%). After excluding two studies [33, 34] with short intervention durations, the yoga group showed significant improvement in the quality of life of patients with knee osteoarthritis compared to the control group, and exhibited lower heterogeneity (SMD = 0.25; 95% CI = 0.03 ~ 0.47; *P* = 0.03; *I*^2^ = 0%), as shown in Fig E in S4 File.

## 4. Discussion

This meta-analysis included 8 RCT studies investigating the effects of Yoga on pain, stiffness, physical function, ADL, and QOL in patients with KOA. Compared to the initial registration plan, we have included three additional secondary outcome measures: physical function, ADL, and QOL. Additionally, we conducted subgroup analyses for the primary outcome measure of pain and sensitivity analyses for other highly heterogeneous outcomes. Our analysis indicates that yoga significantly improves pain, stiffness, and physical function, aligning with previous meta-analytical findings on yoga therapy for OA [40, 41]. Our study shows some differences compared to previous research regarding the im-provement of ADL and QOL in KOA patients through yoga intervention. Previous studies indicated that exercise therapy can effectively improve ADL and QOL in KOA patients [42]. This difference could be attributed to our study focusing solely on yoga as the train-ing type and including only RCTs, leading to variability in the results.

### 4.1 The effect of Yoga on pain

Based on our analysis, we found that yoga can significantly alleviate patients’ perception of pain. Due to the high heterogeneity, we conducted subgroup and sensitivity analyses. In the two studies with an intervention duration >8 weeks, although the intervention duration was longer, yoga training was conducted at home or through online courses, resulting in low patient compliance. For example, in one study, originally planned for three yoga sessions per week, the lack of supervision in online courses led to an average of only 1.9 yoga sessions completed per week by the end of the study[39]. In contrast, in the other four studies with intervention durations ≤8 weeks, yoga training was conducted in activity centers or hospitals, with supervised sessions, resulting in high compliance and completion rates. The disparity in the mode of yoga training may be a contributing factor to the higher heterogeneity observed in these two studies. Based on the analysis results, we believe that supervised, offline yoga classes can effectively enhance patient motivation and compliance, ensure the standardization of yoga poses, and better achieve the expected training volume. This is also an important guarantee for yoga to alleviate pain in patients with KOA.

The pain-alleviating effects of yoga can also be attributed to its ability to reduce inflammation, enhance antioxidative properties, and regulate gene expression. Yoga can alleviate inflammatory responses and reduce the levels of inflammation markers, such as C-reactive protein and interleukin-6 (IL-6), in patients with KOA. This modulation of the immune system and neuroendocrine system helps alleviate symptoms associated with KOA. Patients with KOA often experience increased oxidative stress, where the production of free radicals exceeds the body’s antioxidant capacity [43]. The breathing exercises, stretching movements, and meditation practiced in Yoga are believed to have antioxidative effects [44]. They can reduce oxidative stress and promote normal cellular function. Yoga practice also has the potential to regulate gene expression related to inflammation, immunity, and cell apoptosis. This may contribute to alleviating inflammation and damage caused by KOA [45]. In addition, Yoga practice can also enhance muscle strength around the knee joint, such as quadriceps and hamstring muscles, which increases joint stability and reduces the pressure on the knee joint. This can alleviate pain caused by joint usage to some degree [46]. The movement of the knee joint in yoga poses can help alleviate pain in patients with knee osteoarthritis, increase joint load during walking, and promote the flow of synovial fluid, which can reduce the accumulation of inflammatory substances [47]. In addition, a study by Xu et al. using magnetic resonance imaging observed that joint movement can improve bone marrow lesions in patients with knee osteoarthritis, reducing inflammation and promoting normal bone marrow metabolism [48].

### 4.2 The effect of Yoga on stiffness

Based on our analysis, we found that yoga has a significant effect on improving joint stiffness in patients with KOA, especially after excluding a study that involved training only once a week, which led to a significant reduction in heterogeneity. Therefore, we speculate that the degree of improvement in KOA patients through yoga may be related to the duration of the intervention. Patients with KOA experience degenerative changes in the knee joint cartilage and inflammatory responses, leading to limited options for physical activity [49]. Yoga exercises are characterized by lower intensity and do not require vigorous body positioning or strength training. They focus on slow and gentle movements, which help stretch the limbs and relieve tension in the muscles and soft tissues around the knee joint [50]. Additionally, Yoga incorporates controlled breathing techniques to aid in achieving a state of physical and mental relaxation [51]. This gentle form of exercise is highly suitable for KOA patients as it utilizes slow and controlled movements to increase joint mobility and improve knee joint stiffness [37]. Yoga can also promote blood circulation and lymphatic flow around the knee joint, enhancing the supply of nutrients to the surrounding soft tissues. It improves body self-awareness and attention, helping patients better control muscle tension and alleviate joint stiffness. As a strongly recommended exercise in OA management guidelines, Yoga requires minimal equipment, only requiring a mat or a stable chair to complete [52]. Compared to other land-based exercises and some aerobic activities, Yoga is not only safe but also more comfortable and convenient, with lower requirements for the exercise setting. It gently alleviates knee joint stiffness in patients [18].

### 4.3 The effect of Yoga on physical function

Different types of yoga have beneficial effects on patients with KOA, but with different emphases. The variations in yoga types may also contribute to the heterogeneity observed in our analysis of yoga’s impact on physical functioning improvement. Hatha yoga is suitable for individuals looking to exercise both body and mind comprehensively, aiming to improve symptoms of knee osteoarthritis through the practice of postures, breath control, and meditation [53]. Chair yoga, is more suitable for patients with knee osteoarthritis as it involves low-impact, gentle, and highly adaptable exercises to reduce the burden on the knee joint, increase joint range and flexibility, alleviate pain, enhance function, and com-fort [54].

Not only are there various types of yoga, but each type also has its own advantages in terms of different yoga poses. If conditions permit, training different poses for KOA patients with different disease courses is believed to yield better therapeutic effects. Yoga poses such as Cat-Cow pose and Tree pose can help relax the body, improve balance, and enhance core stability. Meditation practices like Lotus position meditation and Zen meditation can aid in focusing attention, cultivating inner calm, and enhancing self-awareness [55]. Through consistent yoga and meditation practice, individuals can gradually develop a sense of control and confidence in their bodies, reducing tension and anxiety during exercise, thereby promoting overall physical and mental well-being. This may potentially improve exercise phobia and exercise control [56].Choosing a yoga form that suits one’s needs can better help in treating knee osteoarthritis. Among the studies included in this analysis, three did not clearly specify the type of yoga used or involved the combined use of various types of yoga, making it challenging to analyze the specific therapeutic effects of individual yoga practices.

Yoga, as a form of physical activity, benefits patients with knee osteoarthritis in several ways. Through yoga practice, it is possible to strengthen the muscles surrounding the joints, enhancing flexibility and stability, thereby reducing the burden and pressure on the knee joints [39]. Yoga movements engage coordinated muscle groups throughout the body, promoting postural adjustments and optimization, thus minimizing knee joint imbalance and excessive wear. Additionally, yoga’s meditation and breath control help reduce stress and anxiety levels, alleviating the perception of pain. Furthermore, by promoting balance in the neuroendocrine system, yoga can aid in regulating immune function and inflammatory responses, positively impacting the symptoms and progression of knee osteoarthritis. Therefore, as a comprehensive form of physical activity, yoga can contribute to improving the physical function and comfort of knee osteoarthritis patients through multiple mechanisms.

### 4.4 The effect of Yoga on ADL and QOL

The analysis results indicate that Yoga did not significantly ADL and QOL in KOA patients. When analyzing the effect of yoga on the activities of daily living ADL in KOA patients, we found that one study recruited patients with milder symptoms, and at the time of enrollment, their ADL was already relatively good. However, upon excluding this study, we observed a significant improvement in ADL due to yoga.

Since yoga did not show a significant improvement in the QOL for KOA patients, we conducted a sensitivity analysis and found that excluding studies with short treatment duration and low treatment frequency, yoga training showed a significant improvement in QOL for patients with KOA. Therefore, we speculate that increasing the duration of treatment may effectively help improve the QOL for KOA patients. After carefully comparing the included literature and reviewing relevant studies, the following reasons may contribute to these findings. Firstly, the average duration of the included studies was around 8 weeks, which is relatively short. It is possible that such a short duration may not lead to significant changes in the lives and activities of patients, thus failing to capture the potential benefits of long-term Yoga practice for KOA patients. Additionally, the included studies may have used different measurement tools to assess physical function, ADL, and QOL, which could result in differences in the outcomes. This variability is difficult to avoid, as there is still no standardized measurement tool for assessing ADL and QOL in KOA patients to ensure complete consistency of results [57]. It is also unclear whether Yoga can produce the same effects in different stages of KOA. Some studies were conducted in the early stages or milder cases [36, 37], while others may have focused on late-stage or severe cases [34, 35]. Different stages of KOA may have varying impacts on the response to Yoga, which could be a contributing factor to the lack of significant improvement observed.

In summary, Yoga can effectively alleviate pain and stiffness in patients, which already addresses the main concerns of most KOA patients. Furthermore, the improvement in pain and stiffness functions can not only alleviate patients’ emotions but also contribute to enhancing patients’ ADL and QOL. Yoga is still in the experimental stage as a treatment for common clinical diseases such as OA, mental illness and metabolic problems [58–60]. Our analysis provides evidence-based support for the use of Yoga in treating KOA and serves as a reference for clinical trials, promoting Yoga as one of the options for patients and healthcare professionals.

## 5. Study limitations

This review has certain limitations. The included studies in this analysis involved Hatha Yoga and combined training with various Yoga systems. Currently, clinical research is insufficient to support the evaluation of the therapeutic effects of different types of Yoga for individual treatment of KOA patients. Furthermore, the trials were mainly conducted in a few countries, and 37.5% of the studies had sample sizes below 50 cases. However, the average age of each study was over 45 years, which can be used as a reference for the age of Yoga treatment. In order to increase the sample size for analysis, two studies of combination therapy were included, although variables were controlled for (Yoga only), it is unavoidable to determine whether Yoga has a synergistic effect with other treatments. Some studies conducted multiple assessments at different time periods during the treatment process, but we only selected one result that was closer to the mean value of each study for analysis, which may have some impact on the final results.

## 6. Conclusions

After approximately 8 weeks of Yoga treatment for KOA patients, there was a significant reduction in pain, stiffness and physical function, but no significant difference was observed in terms of ADL and QOL compared to the control group. Based on the current evidence analysis, Yoga has shown a good therapeutic effect for KOA by effectively relieving pain, stiffness and physical function. However, further analysis with more sample size is needed to evaluate its impact on ADL and QOL in patients.

## Supporting information

S1 File PRISMA_2020_checklist. (DOCX)

S2 File PubMed search record. (CRDOWNLOAD)

S3 File Excluded literature. (DOC)

S4 File Sensitivity analysis results. (DOC)
