# Supplementary material for: The impact of Yoga on patients with knee osteoarthritis: A systematic review and meta-analysis of randomized controlled trials
Source: PLoS One. 2024 May 16;19(5):e0303641. doi: 10.1371/journal.pone.0303641 (PMC11098307; doi:10.1371/journal.pone.0303641)
Supplement: S3 File — (DOC) [file pone.0303641.s003.doc]

**1.**Nambi GS, Shah AA. Additional effect of iyengar yoga and EMG biofeedback on pain and functional disability in chronic unilateral knee osteoarthritis. Int J Yoga. 2013 Jul;6(2):123-7. doi: 10.4103/0973-6131.113413

**Exclusion reasons:** Control group underwent other training modalities, yoga not considered as a single variable.

**2.** Moonaz SH, Bingham CO 3rd, Wissow L, Bartlett SJ. Yoga in Sedentary Adults with Arthritis: Effects of a Randomized Controlled Pragmatic Trial. J Rheumatol. 2015 Jul;42(7):1194-202. doi: 10.3899/jrheum.141129

**Exclusion reasons:** Included patients were not exclusively those with knee osteoarthritis (KOA).

**3.** Kolasinski SL, Garfinkel M, Tsai AG, Matz W, Van Dyke A, Schumacher HR. Iyengar yoga for treating symptoms of osteoarthritis of the knees: a pilot study. J Altern Complement Med. 2005 Aug;11(4):689-93. doi: 10.1089/acm.2005.11.689

**Exclusion reasons:** Before-after controlled trials not meeting inclusion criteria.

**4.** Ebnezar J, Nagarathna R, Yogitha B, Nagendra HR. Effect of integrated yoga therapy on pain, morning stiffness and anxiety in osteoarthritis of the knee joint: A randomized control study. Int J Yoga. 2012 Jan;5(1):28-36. doi: 10.4103/0973-6131.91708

**Exclusion reasons:** Duplicate publication of the study.

**5.** Deepeshwar S, Tanwar M, Kavuri V, Budhi RB. Effect of Yoga Based Lifestyle Intervention on Patients With Knee Osteoarthritis: A Randomized Controlled Trial. Front Psychiatry. 2018 May 8;9:180. doi: 10.3389/fpsyt.2018.00180

**Exclusion reasons:** Outcome measures did not meet the inclusion criteria.

**6.** Cheung C, Wyman JF, Savik K. Adherence to a Yoga Program in Older Women with Knee Osteoarthritis. J Aging Phys Act. 2016 Apr;24(2):181-8. doi: 10.1123/japa.2015-0048

**Exclusion reasons:** Study was not a randomized controlled trial.

**7.** Bukowski EL, Conway A, Glentz LA, Kurland K, Galantino ML. The effect of iyengar yoga and strengthening exercises for people living with osteoarthritis of the knee: a case series. Int Q Community Health Educ. 2006-2007;26(3):287-305. doi: 10.2190/IQ.26.3.f

**Exclusion reasons:** Case studies, study design not meeting inclusion criteria.

**8.** Brenneman EC, Kuntz AB, Wiebenga EG, Maly MR. A Yoga Strengthening Program Designed to Minimize the Knee Adduction Moment for Women with Knee Osteoarthritis: A Proof-Of-Principle Cohort Study. PLoS One. 2015 Sep 14;10(9):e0136854. doi: 10.1371/journal.pone.0136854

**Exclusion reasons:** Study was not a randomized controlled trial.

**9.** Ebnezar J, Nagarathna R, Yogitha B, Nagendra HR. Effects of an integrated approach of hatha yoga therapy on functional disability, pain, and flexibility in osteoarthritis of the knee joint: a randomized controlled study. J Altern Complement Med. 2012 May;18(5):463-72. doi: 10.1089/acm.2010.0320

**Exclusion reasons:** Duplicate publication of the study.
