# Supplementary material for: The impact of Yoga on patients with knee osteoarthritis: A systematic review and meta-analysis of randomized controlled trials
Source: PLoS One. 2024 May 16;19(5):e0303641. doi: 10.1371/journal.pone.0303641 (PMC11098307; doi:10.1371/journal.pone.0303641)
Supplement: S4 File — (DOC) [file pone.0303641.s004.doc]

**Sensitivity analysis results**

**
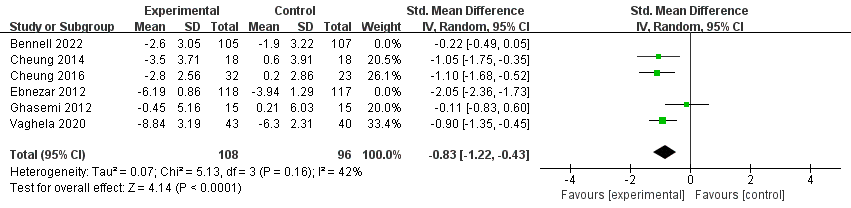
**

**Figure A.Yoga on pain**


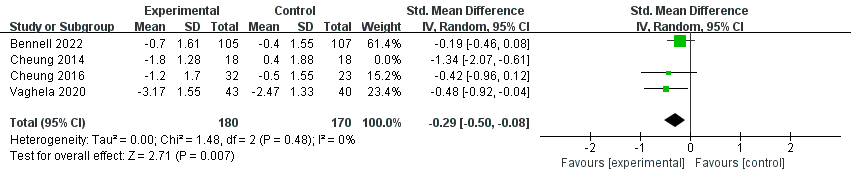


**Figure B. Yoga on stiffness**


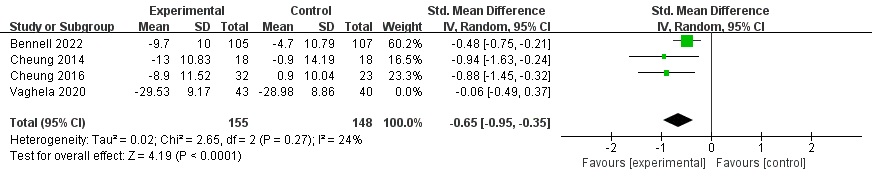


**Figure C. Yoga on physical function**


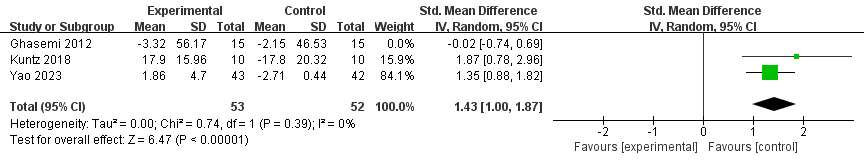


**Figure D. Yoga on ADL outcomes**


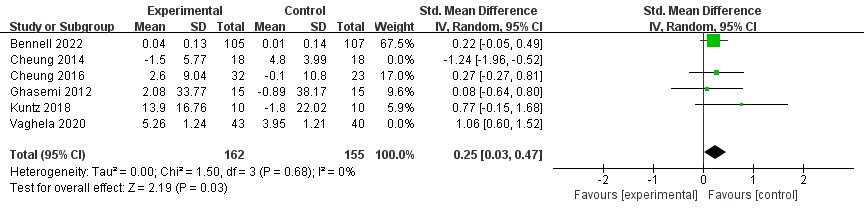


**Figure E.** **Yoga on QOL outcomes**
